# Microbial regulation of offspring diseases mediated by maternal-associated microbial metabolites

**DOI:** 10.3389/fmicb.2022.955297

**Published:** 2022-11-04

**Authors:** Qingru Jiang, Tian Li, Wei Chen, Yingfang Huo, Xiangyu Mou, Wenjing Zhao

**Affiliations:** ^1^Center for Infection and Immunity Studies, School of Medicine, Shenzhen Campus of Sun Yat-sen University, Shenzhen, China; ^2^Department of Gynecology and Obstetrics, The Seventh Affiliated Hospital of Sun Yat-sen University, Shenzhen, China; ^3^Guangdong Provincial Key Laboratory of Colorectal and Pelvic Floor Diseases, The Sixth Affiliated Hospital, Sun Yat-sen University, Guangzhou, China

**Keywords:** short-chain fatty acids (SCFAs), tryptophan derivatives, indole derivatives, branched-chain fatty acids (BCFAs), succinate, bile acid derivatives, trimethylamine-*N*-oxide (TMAO), human milk oligosaccharides (HMOs)

## Abstract

The microbiota plays a crucial role in individuals’ early and long-term health. Previous studies indicated that the microbial regulation of health may start before birth. As the *in utero* environment is (nearly) sterile, the regulation is probably be originated from maternal microbiota and mediated by their metabolites transferred across the placenta. After the birth, various metabolites are continuously delivered to offspring through human milk feeding. Meanwhile, some components, for example, human milk oligosaccharides, in human milk can only be fermented by microbes, which brings beneficial effects on offspring health. Hence, we speculated that human milk-derived metabolites may also play roles in microbial regulation. However, reports between maternal-associated microbial metabolites and offspring diseases are still lacking and sparsely distributed in several fields. Also, the definition of the maternal-associated microbial metabolite is still unclear. Thus, it would be beneficial to comb through the current knowledge of these metabolites related to diseases for assisting our goals of early prediction, early diagnosis, early prevention, or early treatment through actions only on mothers. Therefore, this review aims to present studies showing how researchers came to the path of investigating these metabolites and then to present studies linking them to the development of offspring asthma, type 1 diabetes mellitus, food allergy, neonatal necrotizing enterocolitis, or autism spectrum disorder. Potential English articles were collected from PubMed by searching terms of disease(s), maternal, and a list of microbial metabolites. Articles published within 5 years were preferred.

## Introduction

The microbiota plays a crucial role in individuals’ early and long-term health *via* activating and developing the immune system, developing the central nervous system, and digesting and metabolizing food (Yao et al., 2021). When *in utero*, offspring are protected by maternal immunity and meanwhile they develop their own immunity. The initial development may be partly stimulated by maternal microbiota. Compared with the germ-free group, transiently gestational colonization maternal mice have given birth to germ-free neonates with altered intestinal mucosal innate immune composition and intestinal mucosal transcriptional signatures (Gomez de Agüero et al., 2016). Although the *in utero* environment is sterile or may carry a very tiny number of microbes, various microbial metabolites have been detected in animal umbilical cord blood, placenta, fetal intestine, fetal brain (Vuong et al., 2020; Bi et al., 2021), and human fetal intestine (Li et al., 2020), which sheds us a light that influences of maternal microbiota on fetal health development might be mediated by their metabolites. Macpherson et al. (2017) and Ganal-Vonarburg et al. (2020) have also supported the fact that the development of the fetal immune system was driven by microbial metabolites rather than live micro-organisms.

After birth, mothers can continuously strongly influence their offspring’s health through human milk feeding. In the first 6 months, human milk, containing nutrients, antibodies, bioactive components, microbiota, metabolites, and human milk oligosaccharides (HMOs), fully covers an infant’s needs for nutrition, energy, and protection (Singh et al., 2021). Also, bacterial metabolites in milk, such as short-chain fatty acids (SCFAs), indoles, 12, 13-dihydroxy-9Z-octadecenoic acid (12, 13-DiHOME), and methylamines, have been reported to impact offspring health (Gao et al., 2021; Stinson and Geddes, 2022). As the third most abundant solid component in human milk, HMOs are believed to provide nutrition not to infants but to infant microbiota (Sanchez et al., 2021). Lack of gut bifidobacteria or HMO-utilization genes was related to systemic inflammation in breastfed individuals that have been mitigated by the supplementation of *Bifidobacterium infantis* EVC001 fermenting HMOs into the metabolite indole-3-lactic acid (ILA) (Henrick et al., 2021). Conceptually, the HMOs-derived metabolites probably produced in the infant gut belong to infant metabolites, but HMOs intake by breastfed infants are from human milk, so they can also be considered maternal-associated microbiota metabolites. In general, we could say during pregnancy and lactation stage maternal-associated microbial metabolites may influence offspring health.

Previous studies collectively supported the hypothesis of Developmental Origins of Health and Disease (DOHaD) (Waterland and Michels, 2007). This hypothesis proposes that a critical window during prenatal and postnatal exists for stimuli to influence developmental pathways causing permanent changes in certain diseases’ susceptibility. Since late-postnatal interventions or treatments cannot cure certain diseases but only reduce the syndromes, increasing researchers have speculated that their critical window may appear during very early life, i.e., prenatal stage and lactation stage. For example, supplementation with fish oil during pregnancy has reduced offspring asthma risk (Bisgaard et al., 2016). Current pieces of evidences also supported that maternal diet showed strong impacts on maternal gut microbial composition and further influenced fetal immune development, which was probably mediated by microbial metabolites (Gray et al., 2017; Alsharairi, 2020a).

These metabolites or milk-derived substrates could be detected in maternal blood or human milk, so they are promising indicators or biomarkers to guide us if maternal microbial intervention to regulate offspring diseases is in a good direction or at a sufficient level, which will assist our goal of early prediction, early diagnosis, early prevention, or early treatment through actions only on mothers. And the relevant metabolites from milk-derived substrates will improve our understanding of disease development. But reports between maternal-associated microbial metabolites and offspring diseases are still lacking and sparsely distributed in several fields. Also, the definition of the maternal-associated microbial metabolite is still unclear. Thus, it would be beneficial to comb through the current relevant knowledge for future investigations. We selected immune-mediated, metabolic, and neurodevelopmental diseases, namely asthma, type 1 diabetes mellitus (T1DM), food allergy (FA), neonatal necrotizing enterocolitis (NEC), and autism spectrum disorder (ASD). The exact causes of these diseases are still largely unknown, but both genetic and environmental factors, specifically microbiota, are believed as contributors to their development.

Therefore, this review aims to present studies showing how researchers came to the path of investigating maternal-associated microbial metabolites and then to present studies linking them to the development of offspring asthma, T1DM, FA, NEC, and ASD.

## Search strategy

We identified potential relevant articles from PubMed utilizing the following terms: (asthma/type 1 diabetes/food allergy/necrotizing enterocolitis/autism) AND (maternal) AND (microbiota/microbial/bacterial/fungal metabolite OR microbiota/microbial/bacterial/fungal molecule OR branched-chain fatty acids OR tryptophan OR aromatic amino acid OR other microbial metabolites from the list summarized by Nicholson et al. (2012), except vitamins, glucose, or urea in the list) NOT (review [Publication Type]) AND (English [Language]) (see Supplementary material). The inclusion criteria were original articles, written in English, and published in the last 5 years through 1 September 2022, including mainly animal and human studies. Articles not related to one of the five diseases, maternal, and microbial metabolites/milk-derived substrates were excluded. Additional articles were found from the reference lists of already retrieved publications.

## Asthma

Asthma is one of the most common and non-communicable diseases affecting both children and adults with variable respiratory symptoms and variable airflow limitation, like cough, wheezing, shortness of breath, and chest tightness. The current treatment goals are only to minimize the symptom burden and the risk of adverse asthma outcomes (Ducharme et al., 2014; Papi et al., 2018).

Increasing studies have demonstrated that asthma is associated with the environmental indoor microbiome (Fu et al., 2020; Vandenborght et al., 2021) and human nasopharyngeal-, respiratory-, and gastrointestinal microbiome (Huang, 2015; Teo et al., 2015; Frati et al., 2019; Tang et al., 2021; Lee-Sarwar et al., 2022). However, the addition of antibiotics to the conventional treatment of oral corticosteroids has shown rather limited clinical benefits to control asthma exacerbations in both children and adults (Murray et al., 2021). Antibiotic exposure during childhood can reduce the microbial diversity of the host, increase the risk of asthma development, and prolong the symptoms (Toivonen et al., 2021; Kama et al., 2022). On the contrary, the intervention of probiotic *Lacticaseibacillus rhamnosus* GG (LGG) in infants with a high risk of asthma has gained beneficial effects, although the effects last temporarily (Durack et al., 2018). Recently, Alsharairi (2020b) has summarized that various probiotic strains of specific species of *Bifidobacterium*, *Lactobacillus*, *Bacteroides*, *Enterococcus, Streptococcus, Blautia*, *Ruminococcus*, and *Faecalibacterium prausnitzii* could be strongly impacted by diet and may have the potential to reduce the risk of allergic asthma development. These studies imply that to reduce asthma risk, adding/increasing suitable probiotics or increasing microbial diversity appears more beneficial than erasing microbes without bias.

Recent studies have further indicated that offspring asthma was strongly associated with prenatal micro-organisms exposure. Maternal usage of antibiotics has a dose-related association with offspring asthma risk (Stokholm et al., 2014; Alhasan et al., 2020). Ingestion with probiotic *Bifidobacterium breve* M-16V during pregnancy changed the composition of fecal microbiota in neonates and protected the neonates against allergic airway inflammation accelerated by prenatal exposure to an air pollutant aerosol (Terada-Ikeda et al., 2020). In addition, maternal oral intake of an endotoxin-low lyophilized extract containing multiple toll-like receptor (TLR) ligands derived from a mixture of eight major respiratory tract bacterial pathogens has markedly reduced the susceptibility to allergic airway inflammatory disease in offspring (Mincham et al., 2018).

The above beneficial effects might not directly result from the microbes but be mediated by their microbial products, such as acetate. Acetate is one type of SCFA that is produced by the fermentation of dietary fiber or fermentable fiber, besides other types of fibers and carbohydrates, such as resistant starches or HMOs, collectively termed microbiota-accessible carbohydrates (Gray et al., 2017). The maternal gut microbiota-generated acetate was able to transfer to the fetus across the placenta and led the offspring being more resistant to asthma later in life (Thorburn et al., 2015). Acetate was capable of altering certain gene expressions in the fetal lung, such as the downregulation of gene *Nppa* expression, which suppressed the production of atrial natriuretic peptide, a molecule that participated in epithelial biology and immune regulation. They further demonstrated that maternal intake of a high-fiber diet or acetate protected offspring mice against induced allergic airway disease; however, this effect was failed to see when such intake was given to offspring after birth and throughout lactation. Indirectly, maternal gut bacteria-derived SCFAs, such as butyrate and propionate, separately have shown the ability to reduce stimulation of T lymphocytes *via* regulating dendritic cell function, and then generated a tolerogenic immune feature and enhanced a T-helper 1 (Th1) phenotype protective against asthma development, as asthma is linked to Th2 phenotype dominant (Singh et al., 2010; Singh et al., 2014; Gray et al., 2017). Furthermore, Alsharairi (2020b) has suggested that a very low-calorie ketogenic diet, i.e., an extremely low carbohydrate, high fat, and moderate protein diet, during pregnancy and lactation directly increased the abundance of SCFA-producing microbiota in maternal and infant gut and increased the amount of SCFAs in human milk *via* the entero-mammary pathway, which may contribute to an anti-inflammatory environment and may reduce the risk of infant asthma.

Also, supplementation with fish-oil-derived n-3 long-chain polyunsaturated fatty acids during pregnancy week 22–26 until one week after delivery has led to a 31% reduced risk of asthma in offspring at age of 5 years old (Bisgaard et al., 2016), which may be related to the elevated level of metabolite hydroxy-3-carboxy-4-methyl-5-propyl-2-furanpropanoic acid (hydroxy-CMPF) estimated at age 0 (Rago et al., 2019). The furan fatty acid metabolite CMPFs, a result of microbiome activity, are generally beneficial to human health (Xu et al., 2017) and can be vertically transferred to offspring (Olarini et al., 2022); however, their clinical efficacy and mechanisms in reducing asthma still remain unclear.

Hence, exposure to microbial metabolites, such as acetate and hydroxy-CMPFs, during pregnancy and lactation may be associated with a reduction of offspring asthma risk, whereas more comprehensive studies are required.

## Type 1 diabetes mellitus

Type 1 diabetes mellitus is an autoimmune disease that leads to the destruction of pancreatic β-cells that secret insulin (Lucier and Weinstock, 2022). The exact etiology is still largely unknown. Gene factors have well-explained that individuals with a family history of T1DM are more likely to develop T1DM (Ilonen et al., 2019). However, a large increase in the incidence of T1DM has appeared in children in genetically stable populations (Patterson et al., 2019), which supported that environmental factors may also play etiological roles in its development.

Among the environmental factors, microbiota exposure in recent decades has attracted increasing attention due to their capability to mature the immune system (Ilonen et al., 2019). In longitudinal studies, T1DM patients were associated with a decrease in microbial diversity along with an increased abundance of *Bacteroides, Bifidobacterium pseudocatenulatum, Roseburia hominis, Alistipes shahii*, *Parabacteroides*, *Blautia*, and *Ruminococcus* and with a decreased abundance of *Lactococcus* and *Akkermansia* (Dedrick et al., 2020). And *Bacteroides*-derived lipopolysaccharides have shown notably less ability to activate innate immunity and to elicit endotoxin tolerance than lipopolysaccharides from *Escherichia coli*, a common species in a population with a low incidence of T1DM (Vatanen et al., 2016).

Modification of gut microbiota or ingestion of microbial metabolites might be of interest to prevent T1DM. As an adjunct to insulin therapy, an intake of multispecies probiotics for 6 months has alleviated glycemic levels and inflammatory cytokines in T1DM patients aged 6–18 years old (Wang et al., 2022). The Environmental Determinants of Diabetes in the Young (TEDDY) study has demonstrated that a microbiome with more genes related to fermenting and biosynthesizing SCFAs, such as butyrate, showed protective effects on early-onset human T1DM (Vatanen et al., 2018). In addition, bacterial metabolite acetate or butyrate or consumption of acetate- and butyrate-yielding diet showed the ability to dampen T1DM progression in mouse models (Gao et al., 2009; Sun et al., 2015; Marino et al., 2017; Jacob et al., 2020). However, oral butyrate supplementation has failed to significantly affect innate or adaptive immunity in patients with long-standing T1DM, which may be attributed to differences in details of interventional design or physiology, pathology, and microbiology between humans and animals (de Groot et al., 2020).

Therefore, researchers have speculated that the microbial-related intervention may be better to be performed in early life or even during pregnancy before irreversible influences were formed. Early probiotic intervention only within the age of 27 days has exhibited a 60% reduction in the risk of islet autoimmunity in children in the group with the highest genetic risk of T1DM (Uusitalo et al., 2016). Adjunctive SCFAs, including formate, propionate, and butyrate, administration to maternal rats beginning before pregnancy combined with the administration to offspring after weaning successfully protected against the development of virus-induced T1DM in offspring, but administration to offspring beginning at weaning failed (Needell et al., 2017). Jia et al. (2020) have demonstrated that butyrate treatment during pregnancy and nursing dampened T1DM progression in the female mice offspring, which might be due to butyrate-induced inhibition of the activation of pancreatic dendritic cells in the offspring. Six-week feeding of 1% HMOs that was purified from pooled mature human milk from healthy donors has delayed and reduced T1DM incidence in non-obese diabetic mice and reduced the development of severe pancreatic insulitis in later life along with increased fecal SCFAs (Xiao et al., 2018). Moreover, several clinical studies have investigated whether maternal metabolites in cord blood played roles in offspring T1DM. Specifically, relating to microbial metabolites, lower levels of Trp and succinic acid in cord blood have been associated with later T1DM in the offspring (Oresic et al., 2008). Although the taurine/glycine-conjugated bile acid ratio and kynurenine/tryptophan ratio in cord blood have not been related to offspring T1DM (Vistnes et al., 2018; Tapia et al., 2021), the latter has been linked to carry the T1DM high-risk human leukocyte antigen genotype (heterozygous DQ2/DQ8) (Vistnes et al., 2018).

In summary, exposure to SCFAs, Trp, kynurenine, succinic acid before birth and HMOs-derived metabolites after birth may have influences on T1DM development in offspring.

## Food allergy

Food allergy is defined as “an adverse health effect arising from a specific immune response that occurs reproducibly on exposure to a given food” (Boyce et al., 2010), which can trigger clinical symptoms ranging in severity from mild to life-threatening (Yu et al., 2016). The pathogenesis of FA is still not fully understood, but risk factors include increased hygiene, obesity, nutrition, timing and route of exposure to foods, and microbiome (Sicherer and Sampson, 2018; Childs et al., 2022).

The association between microbiota and FA was investigated only recently. Noval Rivas et al. (2013) first reported that the gut microbiota in mice with food allergy displayed a unique feature and may contribute to the pathogenesis of FA by influencing food antigen-specific regulatory T cells. Thereafter, increasing human cohorts have displayed the possible links between gut microbiota dysbiosis and the pathogenesis of FA (Lee et al., 2020). Probiotic LGG-supplemented formula has been reported to improve tolerance acquisition in infants with cow’s milk allergy, which might be attributed to the increased gut microbial diversity and increased microbial-derived butyrate (Berni Canani et al., 2016). In addition, findings of cohort studies have supported the association between low levels of fecal butyrate at 1 year of age and questionnaire-reported food allergy at 4 years and up to 6 years of age (Sandin et al., 2009; Roduit et al., 2019).

Human milk feeding possesses a long history of being related to reduce FA risk in the general population and high-risk children. But mother’s milk with lower abundance, evenness, and a number of differential bacteria, less butyrate-producing bacteria, high levels of Trp/tyrosine/fatty acid metabolism in the predicted functional pathways of microbiota, or lower levels of butyrate have been related to infants with FA (Stinson et al., 2020; Wang et al., 2021). The concentration of butyrate in mature milk around 0.75 mM may show a protective effect against FA development (Paparo et al., 2021).

The presence of maternal gut commensal genus *Prevotella* may have a protective role in offspring FA development. A lower relative abundance of *Prevotella* has been detected in mothers’ breastmilk of infants with FA (Wang et al., 2021). Moreover, Vuillermin et al. (2020) have suggested that maternal carriage during pregnancy rather than offspring carriage during the infancy of *Prevotella copri* has been positively associated with the absence of offspring FA. Although members of genus *Prevotella* are able to ferment dietary fiber into metabolite SCFA acetate and to ferment fat and fiber into SCFAs and succinate, they have only found an independent relationship between maternal high fat and fiber intake and either fecal succinate or lower risk of offspring FA. Therefore, they have deduced that the reduced risk of offspring FA was mediated not by microbial metabolite SCFA but by succinate during pregnancy. Succinate has been reported to enhance immunity (Rubic et al., 2008; Connors et al., 2018). However, carriage of *P. copri* or augmented fecal and serum succinate levels may associate with diseases, like rheumatoid arthritis, cardiovascular diseases, and inflammatory bowel diseases (Chu et al., 2021; Cui et al., 2021; Fremder et al., 2021). Future studies evaluating the influences of *P. copri* or succinate on offspring FA should also consider their impacts on other relevant diseases.

In summary, butyrate may be a crucial factor in human milk to reduce the risk of offspring FA, and maternal gut *P. copri*-derived succinate may also participate in the regulation of offspring FA risk.

## Neonatal necrotizing enterocolitis

Neonatal necrotizing enterocolitis is one of the most common and devastating acquired diseases in neonates with a spectrum of various intestinal conditions (Neu and Walker, 2011). The pathophysiology is poorly understood; however, the pertinent factors may be prematurity, abnormal microbial colonization, and formula feeding (Alganabi et al., 2019; Quigley et al., 2019; Masi et al., 2021; Huang et al., 2022).

Consistent results have proven that human breast milk-fed infants developed far less NEC than exclusive formula-fed infants did (Miller et al., 2018). This beneficial effect could be derived from HMOs (Bode, 2012). Jantscher-Krenn et al. (2012) have demonstrated that a specific HMO type, disialyllacto-*N*-tetraose (DSLNT), exerted NEC-protective effects. Likewise, in a multicenter clinical cohort study, Autran et al. (2018) analyzed that NEC appeared in infants, who mostly received human milk with a significantly low concentration of DSLNT. Furthermore, Masi et al. (2021) have suggested that 241 nmol/ml could be an optimal threshold level for DSLNT to predict infants to develop NEC (91% accuracy) and to be healthy infants (86% accuracy). Also, intake of milk containing DSLNT below this threshold has exhibited abnormal microbiome development, i.e., with a low abundance of *Bifidobacterium longum* and a high abundance of *Enterobacter cloacae*, which indicated that DSLNT might be prebiotic for *B. longum*. Also, the usage of another two types of HMOs, 2′fucosyllactose and 6′-sialyllactose, alone or in combination showed the capability to inhibit TLR 4 signaling, thus protecting against mice or piglet NEC development (Sodhi et al., 2021). However, the identifications and roles of the above HMOs-derived metabolites in NEC prevention remain unclear.

Branched-chain fatty acids (BCFAs) have been associated with reducing the risk of NEC development and improving intestinal disease conditions (Ran-Ressler et al., 2011; Ran-Ressler et al., 2013). BCFAs are mainly saturated fatty acids with one or more methyl branches on the carbon chain. They could be produced by gut microbes from fermenting branched-chain amino acids. They are major components of the cell membranes of various bacteria species, such as *Lactobacillus* and *Bifidobacterium*, and crucial microbial components in the gastrointestinal tract of neonates. Individuals are greatly exposed to BCFAs during their life *in utero* or breastfeeding period (Ran-Ressler et al., 2008; Jie et al., 2018). In the last month of gestation, the gastrointestinal tract of a full-term infant has been estimated to absorb and metabolize BCFAs from swallowing vernix caseosa (Ran-Ressler et al., 2008); however, the intestinal organs of preterm infants miss such exposure. In addition, preterm-infant human milk contained lower levels BCFAs than term-infant human milk did (Jie et al., 2018). Lack exposure of BCFAs might be a possible reason for a less matured gut in preterm infants.

The effects of SCFAs on NEC development are controversial. The establishment of normal microbiota is delayed in preterm infants, which may result in a deficiency of SCFAs in the gut and further impair the intestinal barrier function (Smith et al., 2013). A recent study has shown that fecal microbiota transplantation with samples from NEC patients resulted in NEC-like intestinal injury in germ-free mice, which may result from the transplanted less-butyrate-producing microbiota downregulating anti-inflammatory regulatory T cells (He et al., 2021). Short-term intervention with probiotic bifidobacteria has reduced the dysbiosis of NEC in extremely preterm infants (gestational age < 28 weeks) and simultaneously increased levels of propionate and butyrate (Athalye-Jape et al., 2022). Also, butyrate in breastmilk has been reported to improve the fetal immature intestinal inflammatory response (Gao et al., 2021; Huang et al., 2022). The above studies have supported that SCFAs may be a new therapeutic agent for NEC. However, SCFAs have been conversely related to intestinal mucosal injury and played a role in the pathogenesis of NEC (Thymann et al., 2009; Roy et al., 2018). Four-week administration of *B. breve* has been effective to promote the establishment of normal intestinal microbial composition in extremely low-birth-weight infants, along with reduced production of butyric acid (Wang et al., 2007). Although the reasons for previous inconsistent results are unclear, the gestational age at birth, postnatal age, microbiota composition, and concentration of metabolites may influence the roles of SCFAs in the gastrointestinal tract. For example, a low concentration of butyrate (2 mM) has enhanced intestinal barrier function and decreased inulin permeability, whereas a high concentration of butyrate (8 mM) has caused severe intestinal epithelial cell apoptosis and disrupted the intestinal barrier (Peng et al., 2007).

Previous studies have mainly focused on investigations of NEC development and possible treatments after birth but until now without leading any effective way to cure it. Lu et al. (2021) have hypothesized that it may be possible to modulate the development of NEC *in utero*. Their mice study has indicated that maternal delivery of aryl hydrocarbon receptor (AHR) ligands, namely tested ligand indole-3-carbinole and “A18,” to the fetus showed the capability to prevent the development of NEC by reducing TLR 4 signaling in the offspring gut. Indole and its derivates are natural AHR ligands and can be transformed into Trp by gut microbiota (Bosi et al., 2020). Through binding them, AHR could be activated and thereby enhance intestinal epithelial barrier function and regulate the immune response in the gut (Gasaly et al., 2021). Similarly, ILA, a metabolite produced by probiotic *B. infantis* fermenting human milk Trp, has also shown anti-inflammatory effects on intestinal epithelial cells by activating AHR (Meng et al., 2020). This anti-inflammatory feature of ILA was found only in the fetal mouse intestines, not in the mature ones. The underlined mechanism remains unclear. Moreover, a recent study has uncovered that not only Trp but also other aromatic amino acids can be metabolized by breastmilk-promoted *Bifidobacterium* species to respective aromatic lactic acids to further impact immune function in early life (Laursen et al., 2021).

In summary, maternal microbial-derived AHR ligands during pregnancy, butyrate, and BCFAs in milk, ILA derived from human milk Trp, and unknown metabolites derived from HMOs may have roles in NEC prevention.

## Autism spectrum disorder

Autism spectrum disorder, also known as autism, is a highly heterogeneous neurodevelopmental disability that is characterized by persistent deficits in social communication and social interaction with the presence of restricted, repetitive patterns of behaviors, interests, or activities (American Psychiatric Association, 2013). Current medical treatments can only mitigate the associated symptoms or co-occurring diagnoses in a short term (Lord et al., 2020). The exact underlying mechanisms of ASD remain unclear, however, both genetic and environmental factors are believed as contributors to its development.

In recent years, the gut microbiota has shown communications with the brain to influence the development/syndromes of diseases like ASD *via* bidirectional brain-gut axis, including the enteric nervous system, immune system, the vagus nerve, aromatic amino acid Trp metabolism, and other various microbial byproducts (Cryan et al., 2019; Chernikova et al., 2021; Needham et al., 2021). Gut microbiota-derived metabolite 4-ethylphenylsulfate (4-EPS) has been indicated to cause ASD-related behavioral abnormalities in naïve wild-type mice (Hsiao et al., 2013; Santamaria et al., 2018). Hsiao et al. (2013) have further demonstrated that oral supplementation with *Bacteroides fragilis* restored the gastrointestinal microbial composition, enhanced the intestinal barrier integrity, reduced the intestinal permeability, improved the leakage of 4-EPS into the bloodstream, completely restored the level of serum metabolite 4-EPS, and finally reduced impairments in communicative, stereotypic, anxiety-like, and sensorimotor behaviors in maternal immune activation offspring who proved features of ASD.

Moreover, increasing reports have suggested that maternal factors, including overweight before pregnancy, prenatal high-fat diet, excessive gestational weight gain, maternal inflammation, and others, are associated with an increased likelihood of offspring ASD (Kim et al., 2017; Windham et al., 2019; Fernandes et al., 2021). Also, recent studies have indicated that maternal microbiota and the associated metabolites could influence fetal neurodevelopment *in utero*. For example, prenatal stress-caused reduction of maternal gut microbiota, including *Parasutterella excrementihominis* and *Bifidobacterium*, dysregulated the metabolic pathways of Trp, a well-studied precursor for multiple metabolites and showing crucial roles in proper immune- and neuro-development, reduced transportation of Trp and its derivative serotonin to fetus across the placenta, and thereby induced aberrant fetal neurodevelopment (Antonson et al., 2020; Chen et al., 2020; Galley et al., 2021). Also, maternal administration of kynurenine, another Trp derivative, has rapidly increased levels of kynurenine in fetal plasma and brain and caused offspring featured with ASD-like behavioral abnormalities (Murakami et al., 2021).

In addition, Vuong et al. (2020) have explored that even without environmental challenges maternal gut microbiota, particularly including *Clostridia*-dominant spore-forming bacteria, were able to restore the thalamocortical axonogenesis in fetuses of antibiotic-treated dams. This was probably mediated by maternal microbiota-derived or -modulated metabolites, such as trimethylamine-*N*-oxide (TMAO) and imidazole propionate, existing both in maternal blood and in the fetal brain. Also, low levels of fecal butyrate and accumulated propionic acid have been reported to be associated with ASD children (Liu et al., 2019; Cotrina et al., 2020). Prenatal administration of propionic acid may contribute to offspring ASD by altering development and behavior during adolescence (Foley et al., 2014). In a clinical screening, untargeted metabolomics in mid-pregnancy maternal serum, bile acid pathways have been associated with offspring ASD (Ritz et al., 2020).

In summary, during pregnancy maternal Trp derivatives, TMAO, imidazole propionate, propionic acid, and bile acid derivatives may participate in the regulation of fetal neurodevelopment diseases, like ASD.

## Discussion

**FIGURE 1:**
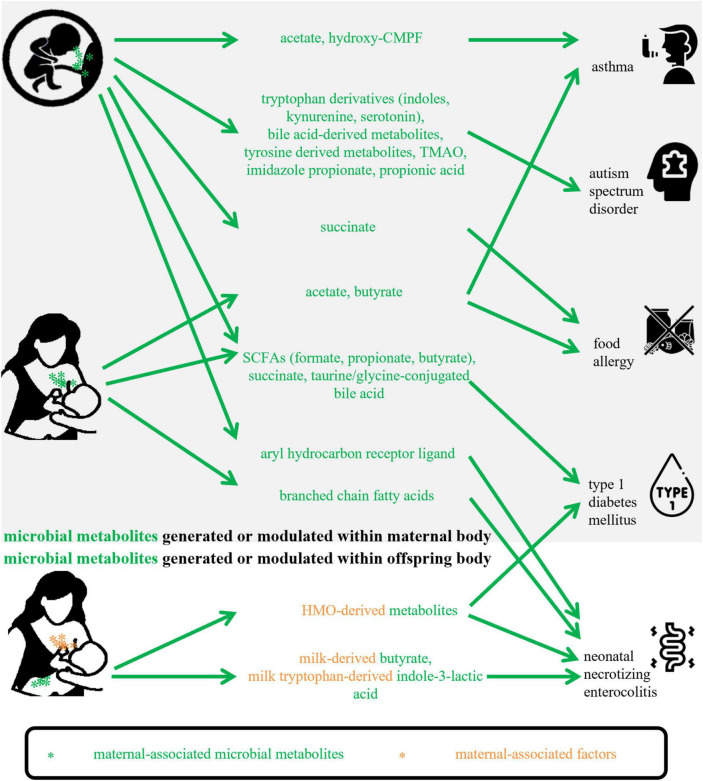
Maternal-associated microbial metabolites generated within maternal body or offspring body in this review. CMPF, 3-carboxy-4-methyl- 5-propyl-2-furanpropanoic acid; TMAO, trimethylamine-N-oxide; SCFAs, short-chain fatty acids; HMO, human milk oligosaccharide.

Micro-organisms and substrates are two basic factors for a microbial metabolite, so in the present review, maternal-associated microbial metabolites can be produced or modulated by maternal microbiota or can be fermented from maternal-derived substrates. Based on collected studies, they could involve metabolites that are generated or modulated (1) within the maternal body and transferred to offspring *via* the placental transportation or breastmilk feeding; (2) within the offspring body in the fermentation of substrates obtained from mothers. Figure 1 shows how the included maternal-associated metabolites in this review can be exposed to offspring. Compared with the metabolites in the former group, substrates-derived metabolites in the latter group and their possible roles were seldomly reported, however, the well-studied substrates, for example, HMOs, in the function of offspring health maintenance will provide a good background for future investigations of their metabolites. About the microbiota obtained from mothers *via* natural birth or human milk feeding, after exposure, they become infant early microbial colonizers, so this kind of metabolite was not included in the present study.

Table 1 shows the main findings of selected publications and lists the maternal-associated metabolites, microbiomes, and substrates or diets. The metabolites included SCFAs, BCFAs, succinate, Trp derivatives, indole derivatives, bile acids derivatives, hydroxy-CMPF, TMAO, and imidazole propionate. Within the limited number of studies, the potential mechanisms of these metabolites on offspring health maintenance may include (1) altering gene expressions encoding molecules that can participate in immune regulation; (2) activating AHR or succinate receptors; (3) enhancing intestinal epithelial barrier function; (4) inhibiting activation of dendritic cells.

**TABLE 1:** Main results of maternal-associated microbial metabolites and offspring diseases mentioned in this review.

Maternal-associated metabolites	Maternal-associated microbiota	Maternal-derived substrate or maternal diet	Study type	Main findings	References
**Asthma**					
Acetate	Maternal gut microbiome	High fiber diet	Mouse model	Placenta-transferred acetate changed the gene expression in the fetal lung that linking to asthma development. Maternal intake of high fiber diet or acetate protected offspring mice against induced allergic airway disease.	Thorburn et al., 2015
Hydroxy-3-carboxy-4-methyl-5-propyl-2-furanpropanoic acid (hydroxy-CMPF)	Not mentioned	Fish oil-derived n-3 long chain polyunsaturated fatty acids (n-3 LCPUFAs)	Cohort study	During pregnancy week 22–26 until 1 week after delivery supplementation with fish oil-derived n-3 LCPUFA reduced 31% risk of asthma in the offspring during the first 5 years of life, and higher level of hydroxy-CMPF in the offspring were estimated at age 0.	Bisgaard et al., 2016; Rago et al., 2019
**Type 1 diabetes mellitus (T1DM)**					
SCFAs (formate, propionate, butyrate)	Not mentioned	Adjunctive SCFAs	Rat model	Adjunctive SCFAs administration to maternal rats beginning prior to pregnancy combined with administration to offspring after weaning successfully protected against development of virus-induced T1DM in offspring, but administration to offspring beginning at weaning failed.	Needell et al., 2017
Butyrate	Not mentioned	Butyrate	Mouse model	Butyrate treatment during pregnancy and nursing dampened T1DM progression in the female mice offspring, which might be due to butyrate-induced inhibition of the activation of pancreatic dendritic cells in the offspring.	Jia et al., 2020
Succinic acid or succinate	Not mentioned	Tryptophan (Trp)	Cohort study	Lower levels of metabolites, including Trp, succinic acid, and creatinine, in cord blood were associated with later T1DM in the offspring.	Oresic et al., 2008
Taurine glycine-conjugated bile acid	Not mentioned	Bile acids	Cohort study	The taurine glycine-conjugated bile acid ratio in cord blood were not able to predict the development of T1DM in the offspring.	Tapia et al., 2021
Kynurenine (Kyn)	Not mentioned	Trp	Cohort study	Kyn Try ratio (KTR) in cord blood plasma at birth were not associated with offspring T1DM, but associated with carrying the T1DM high-risk human leucocyte antigen (HLA) genotype (heterozygous DQ2 DQ8).	Vistnes et al., 2018
SCFAs	Not mentioned	Human milk oligosaccharides (HMOs)	Mouse model	Six-week feeding of 1% HMOs, purified from pooled of mature human milk from healthy donors, delayed and reduced T1DM incidence in non-obese diabetic mice and reduced development of severe pancreatic insulitis in later life along with increased fecal SCFAs.	Xiao et al., 2018
**Food allergy (FA)**					
SCFAs, acetate, butyrate, formate	Not mentioned	Not mentioned	Cohort studies	SCFAs acetate, butyrate, and formate were able to be detected from human milk. And concentrations of acetate and butyrate were lower in atopic mothers than in non-atopic mothers.	Stinson et al., 2020
Butyrate	Microbes from breastmilk	Not mentioned	Case-control study	Microbes from breastmilk showed protective effect against offspring FA partly by producing butyrate.	Wang et al., 2021
Butyrate	Not mentioned	Not mentioned	Cohort study and mouse model	Concentration of butyrate in mature milk around 0.75 mM may show protective effect against offspring FA development.	Paparo et al., 2021
Succinate, SCFAs	*Prevotella copri*	Diet high in fat and fiber	Cohort study	Maternal carriage of *P. copri* during pregnancy may decrease IgE-mediated FA in offspring, especially when women with a diet high in fat and fiber. The protective effect of *P. copri* may be mediated not by SCFAs, but by succinate.	Vuillermin et al., 2020
**Neonatal necrotizing enterocolitis (NEC)**					
Aryl hydrocarbon receptor (AHR) ligand	Not mentioned	Not mentioned	Mouse model	Maternal delivery of AHR ligands, namely ligand indole-3-carbinole and A18 in this study, to the fetus may prevent the development of NEC by reducing toll-like receptor 4 (TLR4) signaling in the offspring gut and independent of leukocyte activation.	Lu et al., 2021
Not mentioned	Not mentioned	HMO	Rat model	A specific HMO type, disialyllacto-N-tetraose (DSLNT), exerted the NEC-protective effects.	Jantscher-Krenn et al., 2012
Not mentioned	Not mentioned	HMO	Cohort study	NEC appeared in infants who mostly received human milk with significantly low concentration of DSLNT.	Autran et al., 2018
Not mentioned	*Bifidobacterium longum* and *Enterobacter cloacae*	HMO	Cohort study	241 nmol ml could be an optimal threshold level for DSLNT to predict infants to develop NEC (91% accuracy) and to be healthy infants (86% accuracy), and also infants obtained milk below this threshold exhibited abnormal microbiome development, i.e., with low abundance of *B. longum* and high abundance of *Enterobacter cloacae*, which indicated DSLNT might be a prebiotic for *B. longum*.	Masi et al., 2021
Not mentioned	Not mentioned	HMO	Mouse and piglet models	The usage of two types of HMOs 2′fucosyllactose and 6′-sialyllactose alone or combination showed capability to inhibit toll-like receptor four signaling, thus protecting against mice or piglet NEC development.	Sodhi et al., 2021
Branched-chain fatty acids (BCFAs)	Not mentioned	Not mentioned	Case-control study	Preterm-infant human milk contained lower levels BCFAs than term-infant human milk did.	Jie et al., 2018
Butyrate	Not mentioned	Breastmilk	*In vitro* and *in vivo* study	Butyrate, as a metabolite of breastmilk complex carbohydrates, improved the fetal immature intestinal inflammatory response induced by cytokine IL-1β.	Gao et al., 2021; Huang et al., 2022
Indole-3-lactic acid (ILA)	*Bifidobacterium infantis*	Milk Trp	*In vivo* study	ILA, a metabolite produced by probiotic *B. infantis* fermenting human milk Trp, has also shown anti-inflammatory effects on intestinal epithelial cells by activating AHR.	Meng et al., 2020
**Autism spectrum disorder (ASD)**					
Serotonin	Not mentioned	Trp	Mouse model	Prenatal stress led to elevated Trp and serotonin in placenta.	Chen et al., 2020
Indoles, Kyn, serotonin	*Parasutterella* and *Bifidobacterium*	Trp	Mouse model	Reductions of Trp-associated microbes and concomitant dysregulation in Trp metabolic machinery in dam and offspring suggested that prenatal stress-induced Trp metabolic dysfunction may mediate aberrant fetal neurodevelopment.	Galley et al., 2021
Kyn	Not mentioned	Trp	Mouse model	Maternal administration of Kyn rapidly increased level of Kyn in fetal plasma and brain, and the offspring mice exhibited behavioral abnormalities similar to those observed in offspring of IL-17A-conditioned mice. IL-17A have been identified as a potential mediator to contribute to the development of ASD.	Murakami et al., 2021
Trimethylamine-*N*-oxide (TMAO) and imidazole propionate	*Clostridia*-dominant spore-forming bacteria	Not mentioned	Mouse model	Without environmental challenges maternal gut microbiota, particularly including *Clostridia*-dominant spore-forming bacteria, were able to restore the thalamocortical axonogenesis in fetus of antibiotic-treated dams	Vuong et al., 2020
Propionic acid (PPA)	Enteric bacteria	Not mentioned	Rat model	Prenatal administration of PPA may contribute to ASD in offspring, altering development and behavior during adolescence.	Foley et al., 2014
Bile acid-derived metabolites	Not mentioned	Bile acids	Case-control study	Bile acid pathways were related to maternal metabolite levels in mid-pregnancy serum associated with ASD in offspring.	Ritz et al., 2020
Not mentioned	*Parasutterella*	Trp, tyrosine, bile acids	Mouse model	Prenatal stressor-exposed mice lost multiple gut metabolic pathways. *Parasutterella* may mediate proper metabolic function in late pregnancy and even early immune development in the offspring.	Antonson et al., 2020

Although plenty of studies have linked microbial SCFAs to several disease preventions or treatments, they were considered to relate to intestinal mucosal injury and contribute to the pathogenesis of NEC, which emphasizes the importance of concentration. It reminds researchers that metabolite concentration is a non-negligible detail when evaluating or interpreting the effects. Also, the relevant studies were mainly about bacterial metabolites and seldom involved fungal or other microbial metabolites.

## Conclusion

Generally, previous researchers have first revealed the possible associations between changes in the microbiota, particularly in the gut, and risks of diverse diseases in the host, and following studies have moved it forward and indicated that the associations may be mediated by the microbial metabolites, especially during pregnancy and lactation maternal-associated microbial metabolites may have crucial roles in participating the microbial regulation of offspring diseases development.

However, studies relevant to the functions of maternal-associated metabolites are still scarce. Before we can carry out metabolite-associated early prediction, early diagnosis, early prevention, or early treatment through actions on mothers, high-quality animal and clinical trials are needed. Also, studies comprehensively evaluating the effects of altered maternal-associated metabolites on overall maternal and infant health maintenance are required.

## Author contributions

All authors conceived, designed, drafted, and revised the manuscript and read and approved the final manuscript.

## Abbreviations

AHR: aryl hydrocarbon receptor
ASD: autism spectrum disorder
BCFAs: branched-chain fatty acids
CMPF: 3-carboxy-4-methyl-5-propyl-2-furanpropanoic acid
DOHaD: developmental origins of health and disease
DSLNT: disialyllacto-*N*-tetraose
FA: food allergy
HMOs: human milk oligosaccharides
ILA: indole-3-lactic acid
LGG: *Lacticaseibacillus rhamnosus* GG
NEC: neonatal necrotizing enterocolitis
SCFAs: short-chain fatty acids
TLR: toll-like receptor
TMAO: trimethylamine-*N*-oxide
Trp: tryptophan
T1DM: type 1 diabetes mellitus.

## References

[B1] Alganabi M. Lee C. Bindi E. Li B. Pierro A. (2019). Recent advances in understanding necrotizing enterocolitis. *F1000Res* 8:107. 10.12688/f1000research.17228.1 30740215 PMC6348433

[B2] Alhasan M. M. Cait A. M. Heimesaat M. M. Blaut M. Klopfleisch R. Wedel A. (2020). Antibiotic use during pregnancy increases offspring asthma severity in a dose-dependent manner. *Allergy* 75 1979–1990. 32064643 10.1111/all.14234

[B3] Alsharairi N. A. (2020a). The infant gut microbiota and risk of asthma: The effect of maternal nutrition during pregnancy and lactation. *Microorganisms* 8:1119. 10.3390/microorganisms8081119 32722458 PMC7466123

[B4] Alsharairi N. A. (2020b). The role of short-chain fatty acids in the interplay between a very low-calorie ketogenic diet and the infant gut microbiota and its therapeutic implications for reducing asthma. *Int. J. Mol. Sci.* 21:9580. 10.3390/ijms21249580 33339172 PMC7765661

[B5] American Psychiatric Association. (2013). . *Diagnostic and statistical manual of mental disorders.* Arlington, VA: American Psychiatric Association.

[B6] Antonson A. M. Evans M. V. Galley J. D. Chen H. J. Rajasekera T. A. Lammers S. M. (2020). Unique maternal immune and functional microbial profiles during prenatal stress. *Sci. Rep.* 10:20288. 10.1038/s41598-020-77265-x 33219314 PMC7679384

[B7] Athalye-Jape G. Esvaran M. Patole S. Simmer K. Nathan E. Doherty D. (2022). Effect of single versus multistrain probiotic in extremely preterm infants: A randomised trial. *BMJ Open Gastroenterol.* 9:e000811. 10.1136/bmjgast-2021-000811 35185013 PMC8860036

[B8] Autran C. A. Kellman B. P. Kim J. H. Asztalos E. Blood A. B. Spence E. C. H. (2018). Human milk oligosaccharide composition predicts risk of necrotising enterocolitis in preterm infants. *Gut* 67 1064–1070. 10.1136/gutjnl-2016-312819 28381523

[B9] Berni Canani R. Sangwan N. Stefka A. T. Nocerino R. Paparo L. Aitoro R. (2016). Lactobacillus rhamnosus GG-supplemented formula expands butyrate-producing bacterial strains in food allergic infants. *ISME J.* 10 742–750. 10.1038/ismej.2015.151 26394008 PMC4817673

[B10] Bi Y. Tu Y. Zhang N. Wang S. Zhang F. Suen G. (2021). Multiomics analysis reveals the presence of a microbiome in the gut of fetal lambs. *Gut* 70 853–864. 10.1136/gutjnl-2020-320951 33589511 PMC8040156

[B11] Bisgaard H. Stokholm J. Chawes B. L. Vissing N. H. Bjarnadottir E. Schoos A. M. (2016). Fish oil-derived fatty acids in pregnancy and wheeze and asthma in offspring. *N. Engl. J. Med.* 375 2530–2539. 10.1056/NEJMoa1503734 28029926

[B12] Bode L. (2012). Human milk oligosaccharides: Every baby needs a sugar mama. *Glycobiology* 22 1147–1162. 10.1093/glycob/cws074 22513036 PMC3406618

[B13] Bosi A. Banfi D. Bistoletti M. Giaroni C. Baj A. (2020). Tryptophan metabolites along the microbiota-gut-brain axis: An interkingdom communication system influencing the gut in health and disease. *Int. J. Tryptophan Res.* 13:1178646920928984. 10.1177/1178646920928984 32577079 PMC7290275

[B14] Boyce J. A. Assa’ad A. Burks A. W. Jones S. M. Sampson H. A. Wood R. A. (2010). Guidelines for the diagnosis and management of food allergy in the United States: Summary of the NIAID-sponsored expert panel report. *J. Allergy Clin. Immunol.* 126 1105–1118. 10.1016/j.jaci.2010.10.008 21134568 PMC4241958

[B15] Chen H. J. Antonson A. M. Rajasekera T. A. Patterson J. M. Bailey M. T. Gur T. L. (2020). Prenatal stress causes intrauterine inflammation and serotonergic dysfunction, and long-term behavioral deficits through microbe- and CCL2-dependent mechanisms. *Transl. Psychiatry* 10:191. 10.1038/s41398-020-00876-5 32546752 PMC7297973

[B16] Chernikova M. A. Flores G. D. Kilroy E. Labus J. S. Mayer E. A. Aziz-Zadeh L. (2021). The brain-gut-microbiome system: Pathways and implications for autism spectrum disorder. *Nutrients* 13:4497. 10.3390/nu13124497 34960049 PMC8704412

[B17] Childs C. E. Munblit D. Ulfman L. Gomez-Gallego C. Lehtoranta L. Recker T. (2022). Potential biomarkers, risk factors, and their associations with IgE-mediated food allergy in early life: A narrative review. *Adv. Nutr.* 13 633–651. 10.1093/advances/nmab122 34596662 PMC8970818

[B18] Chu X. J. Cao N. W. Zhou H. Y. Meng X. Guo B. Zhang H. Y. (2021). The oral and gut microbiome in rheumatoid arthritis patients: A systematic review. *Rheumatology (Oxford)* 60 1054–1066. 10.1093/rheumatology/keaa835 33450018

[B19] Connors J. Dawe N. Van Limbergen J. (2018). The role of succinate in the regulation of intestinal inflammation. *Nutrients* 11:25. 10.3390/nu11010025 30583500 PMC6356305

[B20] Cotrina M. L. Ferreiras S. Schneider P. (2020). High prevalence of self-reported autism spectrum disorder in the propionic acidemia registry. *JIMD Rep.* 51 70–75. 10.1002/jmd2.12083 32071841 PMC7012741

[B21] Cryan J. F. O’riordan K. J. Cowan C. S. M. Sandhu K. V. Bastiaanssen T. F. S. Boehme M. (2019). The microbiota-gut-brain axis. *Physiol. Rev.* 99 1877–2013. 10.1152/physrev.00018.2018 31460832

[B22] Cui H. Chen Y. Li K. Zhan R. Zhao M. Xu Y. (2021). Untargeted metabolomics identifies succinate as a biomarker and therapeutic target in aortic aneurysm and dissection. *Eur. Heart J.* 42 4373–4385. 10.1093/eurheartj/ehab605 34534287 PMC11506060

[B23] de Groot P. F. Nikolic T. Imangaliyev S. Bekkering S. Duinkerken G. Keij F. M. (2020). Oral butyrate does not affect innate immunity and islet autoimmunity in individuals with longstanding type 1 diabetes: A randomised controlled trial. *Diabetologia* 63 597–610. 10.1007/s00125-019-05073-8 31915895

[B24] Dedrick S. Sundaresh B. Huang Q. Brady C. Yoo T. Cronin C. (2020). The role of gut microbiota and environmental factors in type 1 diabetes pathogenesis. *Front. Endocrinol. (Lausanne)* 11:78. 10.3389/fendo.2020.00078 32174888 PMC7057241

[B25] Ducharme F. M. Tse S. M. Chauhan B. (2014). Diagnosis, management, and prognosis of preschool wheeze. *Lancet* 383 1593–1604. 10.1016/S0140-6736(14)60615-2 24792856

[B26] Durack J. Kimes N. E. Lin D. L. Rauch M. Mckean M. Mccauley K. (2018). Delayed gut microbiota development in high-risk for asthma infants is temporarily modifiable by *Lactobacillus* supplementation. *Nat. Commun.* 9:707. 10.1038/s41467-018-03157-4 29453431 PMC5816017

[B27] Fernandes D. J. Spring S. Roy A. R. Qiu L. R. Yee Y. Nieman B. J. (2021). Exposure to maternal high-fat diet induces extensive changes in the brain of adult offspring. *Transl. Psychiatry* 11:149. 10.1038/s41398-021-01274-1 33654064 PMC7925669

[B28] Foley K. A. Ossenkopp K. P. Kavaliers M. Macfabe D. F. (2014). Pre- and neonatal exposure to lipopolysaccharide or the enteric metabolite, propionic acid, alters development and behavior in adolescent rats in a sexually dimorphic manner. *PLoS One* 9:e87072. 10.1371/journal.pone.0087072 24466331 PMC3899377

[B29] Frati F. Salvatori C. Incorvaia C. Bellucci A. Di Cara G. Marcucci F. (2019). The role of the microbiome in asthma: The gut-lung axis. *Int. J. Mol. Sci.* 20:123. 10.3390/ijms20010123 30598019 PMC6337651

[B30] Fremder M. Kim S. W. Khamaysi A. Shimshilashvili L. Eini-Rider H. Park I. S. (2021). A transepithelial pathway delivers succinate to macrophages, thus perpetuating their pro-inflammatory metabolic state. *Cell Rep.* 36:109521. 10.1016/j.celrep.2021.109521 34380041

[B31] Fu X. Norback D. Yuan Q. Q. Li Y. L. Zhu X. H. Hashim J. H. (2020). Indoor microbiome, environmental characteristics and asthma among junior high school students in Johor Bahru, Malaysia. *Environ. Int.* 138:105664. 10.1016/j.envint.2020.105664 32200316

[B32] Galley J. D. Chen H. J. Antonson A. M. Gur T. L. (2021). Prenatal stress-induced disruptions in microbial and host tryptophan metabolism and transport. *Behav. Brain Res.* 414:113471. 10.1016/j.bbr.2021.113471 34280459 PMC9528311

[B33] Ganal-Vonarburg S. C. Hornef M. W. Macpherson A. J. (2020). Microbial-host molecular exchange and its functional consequences in early mammalian life. *Science* 368 604–607. 10.1126/science.aba0478 32381716

[B34] Gao Y. Davis B. Zhu W. Zheng N. Meng D. Walker W. A. (2021). Short-chain fatty acid butyrate, a breast milk metabolite, enhances immature intestinal barrier function genes in response to inflammation in vitro and in vivo. *Am. J. Physiol. Gastrointest. Liver Physiol.* 320 G521–G530. 10.1152/ajpgi.00279.2020 33085904 PMC8238162

[B35] Gao Z. Yin J. Zhang J. Ward R. E. Martin R. J. Lefevre M. (2009). Butyrate improves insulin sensitivity and increases energy expenditure in mice. *Diabetes* 58 1509–1517. 10.2337/db08-1637 19366864 PMC2699871

[B36] Gasaly N. De Vos P. Hermoso M. A. (2021). Impact of bacterial metabolites on gut barrier function and host immunity: A focus on bacterial metabolism and its relevance for intestinal inflammation. *Front. Immunol.* 12:658354. 10.3389/fimmu.2021.658354 34122415 PMC8187770

[B37] Gomez de Agüero M. Ganal-Vonarburg S. C. Fuhrer T. Rupp S. Uchimura Y. Li H. (2016). The maternal microbiota drives early postnatal innate immune development. *Science* 351 1296–1302. 10.1126/science.aad2571 26989247

[B38] Gray L. E. O’hely M. Ranganathan S. Sly P. D. Vuillermin P. (2017). The maternal diet, gut bacteria, and bacterial metabolites during pregnancy influence offspring asthma. *Front. Immunol.* 8:365. 10.3389/fimmu.2017.00365 28408909 PMC5374203

[B39] He Y. Du W. Xiao S. Zeng B. She X. Liu D. (2021). Colonization of fecal microbiota from patients with neonatal necrotizing enterocolitis exacerbates intestinal injury in germfree mice subjected to necrotizing enterocolitis-induction protocol via alterations in butyrate and regulatory T cells. *J. Transl. Med.* 19:510. 10.1186/s12967-021-03109-5 34922582 PMC8684079

[B40] Henrick B. M. Rodriguez L. Lakshmikanth T. Pou C. Henckel E. Arzoomand A. (2021). Bifidobacteria-mediated immune system imprinting early in life. *Cell* 184 3884–3898. 10.1016/j.cell.2021.05.030 34143954

[B41] Hsiao E. Y. Mcbride S. W. Hsien S. Sharon G. Hyde E. R. Mccue T. (2013). Microbiota modulate behavioral and physiological abnormalities associated with neurodevelopmental disorders. *Cell* 155 1451–1463. 10.1016/j.cell.2013.11.024 24315484 PMC3897394

[B42] Huang S. Gao Y. Wang Z. Yang X. Wang J. Zheng N. (2022). Anti-inflammatory actions of acetate, propionate, and butyrate in fetal mouse jejunum cultures ex vivo and immature small intestinal cells in vitro. *Food Sci. Nutr.* 10 564–576. 10.1002/fsn3.2682 35154692 PMC8825721

[B43] Huang Y. J. (2015). The respiratory microbiome and innate immunity in asthma. *Curr. Opin. Pulm. Med.* 21 27–32. 10.1097/Mcp.0000000000000124 25405668 PMC4398309

[B44] Ilonen J. Lempainen J. Veijola R. (2019). The heterogeneous pathogenesis of type 1 diabetes mellitus. *Nat. Rev. Endocrinol.* 15 635–650. 10.1038/s41574-019-0254-y 31534209

[B45] Jacob N. Jaiswal S. Maheshwari D. Nallabelli N. Khatri N. Bhatia A. (2020). Butyrate induced tregs are capable of migration from the GALT to the pancreas to restore immunological tolerance during type-1 diabetes. *Sci. Rep.* 10:19120. 10.1038/s41598-020-76109-y 33154424 PMC7644709

[B46] Jantscher-Krenn E. Zherebtsov M. Nissan C. Goth K. Guner Y. S. Naidu N. (2012). The human milk oligosaccharide disialyllacto-N-tetraose prevents necrotising enterocolitis in neonatal rats. *Gut* 61 1417–1425. 10.1136/gutjnl-2011-301404 22138535 PMC3909680

[B47] Jia L. Cao M. Chen H. Zhang M. Dong X. Ren Z. (2020). Butyrate ameliorates antibiotic-driven type 1 diabetes in the female offspring of nonobese diabetic mice. *J. Agric. Food Chem.* 68 3112–3120. 10.1021/acs.jafc.9b07701 32046486

[B48] Jie L. Qi C. Sun J. Yu R. Q. Wang X. Y. Korma S. A. (2018). The impact of lactation and gestational age on the composition of branched-chain fatty acids in human breast milk. *Food Funct.* 9 1747–1754. 10.1039/c7fo01979c 29497729

[B49] Kama Y. Yamada Y. Koike T. Suzuki K. Enseki M. Hirai K. (2022). Antibiotic treatments prolong the wheezing period in acute exacerbation of childhood bronchial asthma. *Int. Arch. Allergy Immunol* 2022 1–11. 10.1159/000521192 35073552

[B50] Kim S. Kim H. Yim Y. S. Ha S. Atarashi K. Tan T. G. (2017). Maternal gut bacteria promote neurodevelopmental abnormalities in mouse offspring. *Nature* 549 528–532. 10.1038/nature23910 28902840 PMC5870873

[B51] Laursen M. F. Sakanaka M. Von Burg N. Mörbe U. Andersen D. Moll J. M. (2021). Bifidobacterium species associated with breastfeeding produce aromatic lactic acids in the infant gut. *Nat. Microbiol.* 6 1367–1382. 10.1038/s41564-021-00970-4 34675385 PMC8556157

[B52] Lee K. H. Song Y. Wu W. Yu K. Zhang G. (2020). The gut microbiota, environmental factors, and links to the development of food allergy. *Clin. Mol. Allergy* 18:5. 10.1186/s12948-020-00120-x 32265597 PMC7119288

[B53] Lee-Sarwar K. Dedrick S. Momeni B. Kelly R. S. Zeiger R. S. O’connor G. T. (2022). Association of the gut microbiome and metabolome with wheeze frequency in childhood asthma. *J. Allergy Clin. Immunol.* 150 325–336. 10.1016/j.jaci.2022.02.005 35196534 PMC9359927

[B54] Li Y. Toothaker J. M. Ben-Simon S. Ozeri L. Schweitzer R. Mccourt B. T. (2020). In utero human intestine harbors unique metabolome, including bacterial metabolites. *JCI Insight* 5:e138751. 10.1172/jci.insight.138751 33001863 PMC7710283

[B55] Liu S. Li E. Sun Z. Fu D. Duan G. Jiang M. (2019). Altered gut microbiota and short chain fatty acids in Chinese children with autism spectrum disorder. *Sci. Rep.* 9:287. 10.1038/s41598-018-36430-z 30670726 PMC6342986

[B56] Lord C. Brugha T. S. Charman T. Cusack J. Dumas G. Frazier T. (2020). Autism spectrum disorder. *Nat. Rev. Dis. Primers* 6:5. 10.1038/s41572-019-0138-4 31949163 PMC8900942

[B57] Lu P. Yamaguchi Y. Fulton W. B. Wang S. Zhou Q. Jia H. (2021). Maternal aryl hydrocarbon receptor activation protects newborns against necrotizing enterocolitis. *Nat. Commun.* 12:1042. 10.1038/s41467-021-21356-4 33589625 PMC7884836

[B58] Lucier J. Weinstock R. S. (2022). *Diabetes mellitus type 1*. Treasure Island, FL: StatPearls Publishing.

[B59] Macpherson A. J. De Aguero M. G. Ganal-Vonarburg S. C. (2017). How nutrition and the maternal microbiota shape the neonatal immune system. *Nat. Rev. Immunol.* 17 508–517. 10.1038/nri.2017.58 28604736

[B60] Marino E. Richards J. L. Mcleod K. H. Stanley D. Yap Y. A. Knight J. (2017). Gut microbial metabolites limit the frequency of autoimmune T cells and protect against type 1 diabetes. *Nat. Commun.* 18 552–562. 10.1038/ni.3713 28346408

[B61] Masi A. C. Embleton N. D. Lamb C. A. Young G. Granger C. L. Najera J. (2021). Human milk oligosaccharide DSLNT and gut microbiome in preterm infants predicts necrotising enterocolitis. *Gut* 70 2273–2282. 10.1136/gutjnl-2020-322771 33328245 PMC9231288

[B62] Meng D. Sommella E. Salviati E. Campiglia P. Ganguli K. Djebali K. (2020). Indole-3-lactic acid, a metabolite of tryptophan, secreted by *Bifidobacterium longum* subspecies infantis is anti-inflammatory in the immature intestine. *Pediatr. Res.* 88 209–217. 10.1038/s41390-019-0740-x 31945773 PMC7363505

[B63] Miller J. Tonkin E. Damarell R. A. Mcphee A. J. Suganuma M. Suganuma H. (2018). A systematic review and meta-analysis of human milk feeding and morbidity in very low birth weight infants. *Nutrients* 10:707. 10.3390/nu10060707 29857555 PMC6024377

[B64] Mincham K. T. Scott N. M. Lauzon-Joset J. F. Leffler J. Larcombe A. N. Stumbles P. A. (2018). Transplacental immune modulation with a bacterial-derived agent protects against allergic airway inflammation. *J. Clin. Invest.* 128 4856–4869. 10.1172/JCI122631 30153109 PMC6205372

[B65] Murakami Y. Imamura Y. Kasahara Y. Yoshida C. Momono Y. Fang K. (2021). The effects of maternal interleukin-17A on social behavior, cognitive function, and depression-like behavior in mice with altered kynurenine metabolites. *Int. J. Tryptophan Res.* 14:11786469211026639. 10.1177/11786469211026639 34262289 PMC8243115

[B66] Murray C. S. Lucas S. J. Blakey J. Kaplan A. Papi A. Paton J. (2021). A real-life comparative effectiveness study into the addition of antibiotics to the management of asthma exacerbations in primary care. *Eur. Respir. J.* 58:2003599. 10.1183/13993003.03599-2020 33419889

[B67] Needell J. C. Ir D. Robertson C. E. Kroehl M. E. Frank D. N. Zipris D. (2017). Maternal treatment with short-chain fatty acids modulates the intestinal microbiota and immunity and ameliorates type 1 diabetes in the offspring. *PLoS One* 12:e0183786. 10.1371/journal.pone.0183786 28886045 PMC5590848

[B68] Needham B. D. Adame M. D. Serena G. Rose D. R. Preston G. M. Conrad M. C. (2021). Plasma and fecal metabolite profiles in autism spectrum disorder. *Biol. Psychiatry* 89 451–462. 10.1016/j.biopsych.2020.09.025 33342544 PMC7867605

[B69] Neu J. Walker W. A. (2011). Medical progress: Necrotizing enterocolitis. *N. Engl. J. Med.* 364 255–264. 10.1056/NEJMra1005408 21247316 PMC3628622

[B70] Nicholson J. K. Holmes E. Kinross J. Burcelin R. Gibson G. Jia W. (2012). Host-gut microbiota metabolic interactions. *Science* 336 1262–1267. 10.1126/science.1223813 22674330

[B71] Noval Rivas M. Burton O. T. Wise P. Zhang Y. Q. Hobson S. A. Garcia Lloret M. (2013). A microbiota signature associated with experimental food allergy promotes allergic sensitization and anaphylaxis. *J. Allergy Clin. Immunol.* 131 201–212. 10.1016/j.jaci.2012.10.026 23201093 PMC3860814

[B72] Olarini A. Ernst M. Gürdeniz G. Kim M. Brustad N. Bønnelykke K. (2022). Vertical transfer of metabolites detectable from newborn’s dried blood spot samples ssing UPLC-MS: A chemometric study. *Metabolites* 12:94. 10.3390/metabo12020094 35208170 PMC8879569

[B73] Oresic M. Simell S. Sysi-Aho M. Nanto-Salonen K. Seppanen-Laakso T. Parikka V. (2008). Dysregulation of lipid and amino acid metabolism precedes islet autoimmunity in children who later progress to type 1 diabetes. *J. Exp. Med.* 205 2975–2984. 10.1084/jem.20081800 19075291 PMC2605239

[B74] Paparo L. Nocerino R. Ciaglia E. Di Scala C. De Caro C. Russo R. (2021). Butyrate as a bioactive human milk protective component against food allergy. *Allergy* 76 1398–1415. 10.1111/all.14625 33043467 PMC8247419

[B75] Papi A. Brightling C. Pedersen S. E. Reddel H. K. (2018). Asthma. *Lancet* 391 783–800. 10.1016/S0140-6736(17)33311-1 29273246

[B76] Patterson C. C. Harjutsalo V. Rosenbauer J. Neu A. Cinek O. Skrivarhaug T. (2019). Trends and cyclical variation in the incidence of childhood type 1 diabetes in 26 European centres in the 25 year period 1989-2013: A multicentre prospective registration study. *Diabetologia* 62 408–417. 10.1007/s00125-018-4763-3 30483858

[B77] Peng L. He Z. Chen W. Holzman I. R. Lin J. (2007). Effects of butyrate on intestinal barrier function in a Caco-2 cell monolayer model of intestinal barrier. *Pediatr. Res.* 61 37–41. 10.1203/01.pdr.0000250014.92242.f3 17211138

[B78] Quigley M. Embleton N. D. Mcguire W. (2019). Formula versus donor breast milk for feeding preterm or low birth weight infants. *Cochrane Database Syst. Rev.* 7:CD002971. 10.1002/14651858.CD002971.pub5 31322731 PMC6640412

[B79] Rago D. Rasmussen M. A. Lee-Sarwar K. A. Weiss S. T. Lasky-Su J. Stokholm J. (2019). Fish-oil supplementation in pregnancy, child metabolomics and asthma risk. *EBioMedicine* 46 399–410. 10.1016/j.ebiom.2019.07.057 31399385 PMC6712349

[B80] Ran-Ressler R. R. Devapatla S. Lawrence P. Brenna J. T. (2008). Branched chain fatty acids are constituents of the normal healthy newborn gastrointestinal tract. *Pediatr. Res.* 64 605–609. 10.1203/PDR.0b013e318184d2e6 18614964 PMC2662770

[B81] Ran-Ressler R. R. Glahn R. P. Bae S. Brenna J. T. (2013). Branched chain fatty acids in the neonatal gut and estimated dietary intake in infancy and adulthood. *Nestle Nutr. Inst. Workshop Ser.* 77 133–143. 10.1159/000351396 24107503 PMC4029774

[B82] Ran-Ressler R. R. Khailova L. Arganbright K. M. Adkins-Rieck C. K. Jouni Z. E. Koren O. (2011). Branched chain fatty acids reduce the incidence of necrotizing enterocolitis and alter gastrointestinal microbial ecology in a neonatal rat model. *PLoS One* 6:e29032. 10.1371/journal.pone.0029032 22194981 PMC3237582

[B83] Ritz B. Yan Q. Uppal K. Liew Z. Cui X. Ling C. (2020). Untargeted metabolomics screen of mid-pregnancy maternal serum and autism in offspring. *Autism Res.* 13 1258–1269. 10.1002/aur.2311 32496662 PMC13292088

[B84] Roduit C. Frei R. Ferstl R. Loeliger S. Westermann P. Rhyner C. (2019). High levels of butyrate and propionate in early life are associated with protection against atopy. *Allergy* 74 799–809. 10.1111/all.13660 30390309

[B85] Roy S. K. Meng Q. Sadowitz B. D. Kollisch-Singule M. Yepuri N. Satalin J. (2018). Enteral administration of bacteria fermented formula in newborn piglets: A high fidelity model for necrotizing enterocolitis (NEC). *PLoS One* 13:e0201172. 10.1371/journal.pone.0201172 30036384 PMC6056052

[B86] Rubic T. Lametschwandtner G. Jost S. Hinteregger S. Kund J. Carballido-Perrig N. (2008). Triggering the succinate receptor GPR91 on dendritic cells enhances immunity. *Nat. Immunol.* 9 1261–1269. 10.1038/ni.1657 18820681

[B87] Sanchez C. Fente C. Regal P. Lamas A. Lorenzo M. P. (2021). Human milk oligosaccharides (HMOs) and infant microbiota: A scoping review. *Foods* 10:1429. 10.3390/foods10061429 34203072 PMC8234547

[B88] Sandin A. Braback L. Norin E. Bjorksten B. (2009). Faecal short chain fatty acid pattern and allergy in early childhood. *Acta Paediatr.* 98 823–827. 10.1111/j.1651-2227.2008.01215.x 19173682

[B89] Santamaria L. Reveron I. De Felipe F. L. De Las Rivas B. Munoz R. (2018). Ethylphenol formation by *Lactobacillus plantarum*: Identification of the enzyme involved in the reduction of vinylphenols. *Appl. Environ. Microbiol.* 84 e1064–18. 10.1128/AEM.01064-18 29934329 PMC6102998

[B90] Sicherer S. H. Sampson H. A. (2018). Food allergy: A review and update on epidemiology, pathogenesis, diagnosis, prevention, and management. *J. Allergy Clin. Immunol.* 141 41–58. 10.1016/j.jaci.2017.11.003 29157945

[B91] Singh K. S. Singh B. P. Rokana N. Singh N. Kaur J. Singh A. (2021). Bio-therapeutics from human milk: Prospects and perspectives. *J. Appl. Microbiol.* 131 2669–2687. 10.1111/jam.15078 33740837

[B92] Singh N. Gurav A. Sivaprakasam S. Brady E. Padia R. Shi H. (2014). Activation of Gpr109a, receptor for niacin and the commensal metabolite butyrate, suppresses colonic inflammation and carcinogenesis. *Immunity* 40 128–139. 10.1016/j.immuni.2013.12.007 24412617 PMC4305274

[B93] Singh N. Thangaraju M. Prasad P. D. Martin P. M. Lambert N. A. Boettger T. (2010). Blockade of dendritic cell development by bacterial fermentation products butyrate and propionate through a transporter (Slc5a8)-dependent inhibition of histone deacetylases. *J. Biol. Chem.* 285 27601–27608. 10.1074/jbc.M110.102947 20601425 PMC2934627

[B94] Smith P. M. Howitt M. R. Panikov N. Michaud M. Gallini C. A. Bohlooly Y. M. (2013). The microbial metabolites, short-chain fatty acids, regulate colonic treg cell homeostasis. *Science* 341 569–573. 10.1126/science.1241165 23828891 PMC3807819

[B95] Sodhi C. P. Wipf P. Yamaguchi Y. Fulton W. B. Kovler M. Nino D. F. (2021). The human milk oligosaccharides 2’-fucosyllactose and 6’-sialyllactose protect against the development of necrotizing enterocolitis by inhibiting toll-like receptor 4 signaling. *Pediatr. Res.* 89 91–101. 10.1038/s41390-020-0852-3 32221473 PMC7529714

[B96] Stinson L. F. Geddes D. T. (2022). Microbial metabolites: The next frontier in human milk. *Trends Microbiol.* 30 408–410. 10.1016/j.tim.2022.02.007 35282976

[B97] Stinson L. F. Gay M. C. L. Koleva P. T. Eggesbø M. Johnson C. C. Wegienka G. (2020). Human milk from atopic mothers has lower levels of short chain fatty acids. *Front. Immunol.* 11:1427. 10.3389/fimmu.2020.01427 32903327 PMC7396598

[B98] Stokholm J. Sevelsted A. Bennelykke K. Bisgaard H. (2014). Maternal propensity for infections and risk of childhood asthma: A registry-based cohort study. *Lancet Respir. Med.* 2 631–637. 10.1016/S2213-2600(14)70152-3 25066330

[B99] Sun J. Furio L. Mecheri R. Van Der Does A. M. Lundeberg E. Saveanu L. (2015). Pancreatic beta-cells limit autoimmune diabetes via an immunoregulatory antimicrobial peptide expressed under the influence of the gut microbiota. *Immunity* 43 304–317. 10.1016/j.immuni.2015.07.013 26253786

[B100] Tang H. H. F. Lang A. Teo S. M. Judd L. M. Gangnon R. Evans M. D. (2021). Developmental patterns in the nasopharyngeal microbiome during infancy are associated with asthma risk. *J. Allergy Clin. Immunol.* 147 1683–1691. 10.1016/j.jaci.2020.10.009 33091409 PMC7571460

[B101] Tapia G. Suvitaival T. Ahonen L. Lund-Blix N. A. Njølstad P. R. Joner G. (2021). Prediction of type 1 diabetes at birth: Cord blood metabolites vs genetic risk score in the norwegian mother, father, and child cohort. *J. Clin. Endocrinol. Metab.* 106 e4062–e4071. 10.1210/clinem/dgab400 34086903 PMC8475222

[B102] Teo S. M. Mok D. Pham K. Kusel M. Serralha M. Troy N. (2015). The infant nasopharyngeal microbiome impacts severity of lower respiratory infection and risk of asthma development. *Cell Host Microbe* 17 704–715. 10.1016/j.chom.2015.03.008 25865368 PMC4433433

[B103] Terada-Ikeda C. Kitabatake M. Hiraku A. Kato K. Yasui S. Imakita N. (2020). Maternal supplementation with *Bifidobacterium breve* M-16V prevents their offspring from allergic airway inflammation accelerated by the prenatal exposure to an air pollutant aerosol. *PLoS One* 15:e0238923. 10.1371/journal.pone.0238923 32915886 PMC7485856

[B104] Thorburn A. N. Mckenzie C. I. Shen S. Stanley D. Macia L. Mason L. J. (2015). Evidence that asthma is a developmental origin disease influenced by maternal diet and bacterial metabolites. *Nat. Commun.* 6:7320. 10.1038/ncomms8320 26102221

[B105] Thymann T. Moller H. K. Stoll B. Stoy A. C. F. Buddington R. K. Bering S. B. (2009). Carbohydrate maldigestion induces necrotizing enterocolitis in preterm pigs. *Am. J. Physiol. Gastrointest. Liver Physiol.* 297 G1115–G1125. 10.1152/ajpgi.00261.2009 19808655 PMC2850085

[B106] Toivonen L. Schuez-Havupalo L. Karppinen S. Waris M. Hoffman K. L. Camargo C. A. (2021). Antibiotic treatments during infancy, changes in nasal microbiota, and asthma development: Population-based cohort study. *Clin. Infect. Dis.* 72 1546–1554. 10.1093/cid/ciaa262 32170305 PMC8096219

[B107] Uusitalo U. Liu X. Yang J. Aronsson C. A. Hummel S. Butterworth M. (2016). Association of early exposure of probiotics and islet autoimmunity in the TEDDY study. *JAMA Pediatr.* 170 20–28. 10.1001/jamapediatrics.2015.2757 26552054 PMC4803028

[B108] Vandenborght L. E. Enaud R. Urien C. Coron N. Girodet P. O. Ferreira S. (2021). Type 2-high asthma is associated with a specific indoor mycobiome and microbiome. *J. Allergy Clin. Immunol.* 147 1296–1305. 10.1016/j.jaci.2020.08.035 32926879 PMC7486598

[B109] Vatanen T. Franzosa E. A. Schwager R. Tripathi S. Arthur T. D. Vehik K. (2018). The human gut microbiome in early-onset type 1 diabetes from the TEDDY study. *Nature* 562 589–594. 10.1038/s41586-018-0620-2 30356183 PMC6296767

[B110] Vatanen T. Kostic A. D. D’hennezel E. Siljander H. Franzosa E. A. Yassour M. (2016). Variation in microbiome LPS immunogenicity contributes to autoimmunity in humans. *Cell* 165 842–853. 10.1016/j.cell.2016.04.007 27133167 PMC4950857

[B111] Vistnes M. Tapia G. Mårild K. Midttun Ø Ueland P. M. Viken M. K. (2018). Plasma immunological markers in pregnancy and cord blood: A possible link between macrophage chemo-attractants and risk of childhood type 1 diabetes. *Am. J. Reprod. Immunol.* 79 e12802. 10.1111/aji.12802 29266506

[B112] Vuillermin P. J. O’hely M. Collier F. Allen K. J. Tang M. L. K. Harrison L. C. (2020). Maternal carriage of prevotella during pregnancy associates with protection against food allergy in the offspring. *Nat. Commun.* 11:1452. 10.1038/s41467-020-14552-1 32210229 PMC7093478

[B113] Vuong H. E. Pronovost G. N. Williams D. W. Coley E. J. L. Siegler E. L. Qiu A. (2020). The maternal microbiome modulates fetal neurodevelopment in mice. *Nature* 586 281–286. 10.1038/s41586-020-2745-3 32968276 PMC7554197

[B114] Wang C. H. Yen H. R. Lu W. L. Ho H. H. Lin W. Y. Kuo Y. W. (2022). Adjuvant probiotics of *Lactobacillus salivarius* subsp. salicinius AP-32, L. johnsonii MH-68, and *Bifidobacterium animalis* subsp. lactis CP-9 attenuate glycemic levels and inflammatory cytokines in patients with type 1 diabetes mellitus. *Front. Endocrinol. (Lausanne)* 13:754401. 10.3389/fendo.2022.754401 35299968 PMC8921459

[B115] Wang C. Shoji H. Sato H. Nagata S. Ohtsuka Y. Shimizu T. (2007). Effects of oral administration of *Bifidobacterium breve* on fecal lactic acid and short-chain fatty acids in low birth weight infants. *J. Pediatr. Gastroenterol. Nutr.* 44 252–257. 10.1097/01.mpg.0000252184.89922.5f 17255840

[B116] Wang S. Wei Y. Liu L. Li Z. (2021). Association between breastmilk microbiota and food allergy in infants. *Front. Cell. Infect. Microbiol.* 11:770913. 10.3389/fcimb.2021.770913 35096637 PMC8790183

[B117] Waterland R. A. Michels K. B. (2007). Epigenetic epidemiology of the developmental origins hypothesis. *Annu. Rev. Nutr.* 27 363–388. 10.1146/annurev.nutr.27.061406.093705 17465856

[B118] Windham G. C. Anderson M. Lyall K. Daniels J. L. Kral T. V. E. Croen L. A. (2019). Maternal pre-pregnancy body mass index and gestational weight gain in relation to autism spectrum disorder and other developmental disorders in offspring. *Autism Res.* 12 316–327. 10.1002/aur.2057 30575327 PMC7778460

[B119] Xiao L. Van’t Land B. Engen P. A. Naqib A. Green S. J. Nato A. (2018). Human milk oligosaccharides protect against the development of autoimmune diabetes in NOD-mice. *Sci. Rep.* 8:3829. 10.1038/s41598-018-22052-y 29497108 PMC5832804

[B120] Xu L. Sinclair A. J. Faiza M. Li D. M. Han X. L. Yin H. Y. (2017). Furan fatty acids - beneficial or harmful to health? *Prog. Lipid Res.* 68 119–137. 10.1016/j.plipres.2017.10.002 29051014

[B121] Yao Y. Cai X. Ye Y. Wang F. Chen F. Zheng C. (2021). The role of microbiota in infant health: From early life to adulthood. *Front. Immunol.* 12:708472. 10.3389/fimmu.2021.708472 34691021 PMC8529064

[B122] Yu W. Freeland D. M. H. Nadeau K. C. (2016). Food allergy: Immune mechanisms, diagnosis and immunotherapy. *Nat. Rev. Immunol.* 16 751–765. 10.1038/nri.2016.111 27795547 PMC5123910

