# Supplementary material for: Microbial regulation of offspring diseases mediated by maternal-associated microbial metabolites
Source: Front Microbiol. 2022 Nov 4;13:955297. doi: 10.3389/fmicb.2022.955297 (PMC9672376; doi:10.3389/fmicb.2022.955297)
Supplement: Supplementary file 1 [file Table_1.DOCX]

Search items in PubMed

((asthma) or (type 1 diabetes) or (food allergy) or (necrotizing enterocolitis) or (autism)) AND (maternal) AND (#1) NOT (review [Publication Type]) AND (English [Language])

#1:

(microbiota metabolite) OR (microbial metabolite) OR (bacterial metabolite) OR (fungal metabolite) OR (bacterial molecule) OR (microbial molecule) OR (fungal molecule) OR (short-chain fatty acids) or (acetate) or (propionate) or (butyrate) or (isobutyrate) or (2-methylpropionate) or (valerate) or (isovalerate) or (hexanoate) or (bile acids) or (cholate) or (hyocholate) or (deoxycholate) or (chenodeoxycholate) or (taurocholate) or (glycocholate) or (taurochenoxycholate) or (glycochenodeoxycholate) or (taurocholate) or (lithocholate) or (ursodeoxycholate) or (hyodeoxycholate) or (taurohyocholate) or (choline metabolites) or (methylamine) or (dimethylamine) or (trimethylamine) or (trimethylamine-N-oxide) or (dimethylglycine) or (betaine) or (phenolic derivatives) or (benzoyl derivatives) or (phenyl derivatives) or (benzoic acid) or (hippuric acid) or (2-hydroxyhippuric acid) or (2-hydroxybenzoic acid) or (3-hydroxyhippuric acid) or (3-hydroxybenzoic acid) or (4-hydroxybenzoic acid) or (3-hydroxyphenylpropionate) or (4-hydroxyphenylpropionate) or (3-hydroxycinnamate) or (4-methylphenol) or (tyrosine) or (phenylalanine) or (4-cresol) or (4-cresyl sulfate) or (4-cresyl) or (glucuronide) or (4-hydroxyphenylacetate) or (3,4-dihydroxyphenylacetate) or (phenylacetylglycine) or (phenylacetylglutamine) or (phenylacetylglycine) or (phenylacetate) or (phenylpropionate) or (phenylpropionylglycine) or (cinnamoylglycine) or (indole derivatives) or (N-acetyltryptophan) or (indoleacetate) or (indoleacetylglycine (IAG)) or (indole) or (indoxyl sulfate) or (indole-3-propionate) or (melatonin) or (melatonin) or (6-sulfate) or (serotonin) or (5-hydroxyindole) or (Polyamines) or (putrescine) or (cadaverine) or (spermidine) or (spermine) or (conjugated fatty acids) or (peptidoglycan) or (acylglycerols) or (sphingomyelin) or (cholesterol) or (phosphatidylcholines) or (phosphoethanolamines) or (triglycerides) or (D-lactate) or (formate) or (methanol) or (ethanol) or (succinate) or (lysine) or (a-ketoisovalerate) or (creatine) or (creatinine) or (endocannabinoids) or (2-arachidonoylglycerol) or (N-arachidonoylethanolamide) or (lipopolysaccharide) or (branched chain fatty acids) or (tryptophan) OR (aromatic amino acid)
